# Exploring the potential for advanced nursing practice role development in Kenya: a qualitative study

**DOI:** 10.1186/s12912-014-0033-y

**Published:** 2014-11-14

**Authors:** Linda Anne East, John Arudo, Martha Loefler, Catrin Mai Evans

**Affiliations:** School of Health Sciences, South Block Link, Queens Medical Centre, The University of Nottingham, Nottingham, NG7 2HA UK; Masinde Muliro University of Science and Technology, Kakamega, Kenya; Aga Khan University, Nairobi, Kenya

**Keywords:** Advanced nursing practice, Sub-Saharan Africa, Task-shifting, Nursing workforce, Qualitative research

## Abstract

**Background:**

Definitions of advanced nursing practice abound, yet little has been published concerning the context for advanced nursing in sub-Saharan Africa. This study set out to explore the existence of, and potential for, advanced nursing practice in Kenya.

**Methods:**

Ten nurses were invited to participate in semi-structured qualitative interviews. Participants were purposively selected to provide insight into the practice of experienced nurses in urban, rural, community, hospital, public and private health care settings. Interview narratives were recorded, transcribed and subsequently analysed using a thematic approach.

**Results:**

All participants reported that they were engaged in the delivery of expert, evidence-based care. The majority also undertook administrative activities, teaching in the practice area and policy and practice advocacy. However, only the two private practice nurses interviewed during the study were working with the level of autonomy that might be expected of advanced nurse practitioners.

**Conclusions:**

While participants were undertaking many of the activities associated with advanced nursing roles, advanced nursing practice as widely understood in the (largely western derived) international literature was not identified. The nurses practicing with the greatest autonomy were generally those with the lowest educational qualifications rather than the highest. Highly qualified nurses and midwives tend to move into management and education, and see little opportunity for advancement while remaining in clinical practice. It is notable that, although a growing number of universities offer master’s level education, no African countries have yet regulated an advanced level of practice. The existence of the physician substitute ‘clinical officer’ cadre in Kenya, as in other Sub-Saharan African countries, suggests that the development of the advanced nurse practitioner role is unlikely at present. However, there is a pressing need for advanced nurses and midwives who can implement evidence-based practice and exercise clinical leadership in the drive to attain the Millennium Development Goals and their post-2015 successors.

## Introduction

This paper presents the findings from a small-scale scoping project that aimed to explore the nature of, and potential for, advanced nursing practice in Kenya. The project was carried out as part of an existing, European Union-funded collaboration between the Aga Khan University, East Africa and the University of Nottingham, United Kingdom entitled ‘*Improving Nursing Education and Practice in East Africa’* (INEPEA). INEPEA worked with universities in Kenya, Uganda and Tanzania to develop a shared framework for a master’s level curriculum designed to support advanced and specialist nursing practice in the region. The study reported here was conducted as a follow up to an INEPEA situation analysis survey that identified the need for more detailed examination of existing senior nursing roles in Kenya, with reference to internationally recognised frameworks for advanced practice. Further details of the INEPEA project and its outcomes can be found online in its final report [1].

## Background

According to the International Council of Nurses (ICN), an Advanced Practice Nurse (APN) is:

A registered nurse who has acquired the expert knowledge base, complex decision-making skills and clinical competencies for advanced practice, the characteristics of which are shaped by the context and/or country in which s/he is credentialed. A masters degree is recommended for entry level [2].

The ICN’s definition emphasises the knowledge and expertise of the APN, which is derived from both theory and experience. Contextual variation in APN roles is acknowledged, but a master’s degree is recommended for educational preparation. In general, the APN title embraces the roles of both nurse practitioner and clinical nurse specialist, hence ‘APN’ will be used as the umbrella term throughout this paper.

APN roles have their roots in healthcare developments in the United States [3]. The key drivers for APN role development in many countries has been medical substitution alongside a strong professionalising impetus in which enhanced status, recognition and remuneration is accorded to nursing roles through a formalised process of regulation and educational preparation [4]. In many cases, the substitution agenda is clear, but the nursing profession has had only mixed success in creating recognition for an enhanced nursing role through formalised regulation, education and the development of new career pathways [5].

In a survey of international developments in advanced nursing practice, Schober and Affara [6] note there is little evidence documenting the development of recognized APN roles in Sub-Saharan African countries. However, many African countries are increasingly embracing policies of ‘task-shifting’, or the delegating of tasks upwards or downwards through the perceived hierarchies of the health professions in order to address health workforce shortages [7]. There is a burgeoning literature on task shifting and, indeed, a growing evidence base demonstrating comparable health outcomes for doctor or nurse led care in Sub-Saharan Africa [8]. It is interesting to note, however, that task shifting/medical substitution debates in Africa appear to be developing quite independently of debates and discourses on advanced nursing practice despite the potential overlaps between the two agendas [3,9]. A recent survey of nursing and midwifery regulatory reform in east, central and southern Africa concluded that there is a lack of alignment between the regulatory environment and the expanding role of nurses and midwives [10].

Indeed, to date, it seems that no African countries have regulated an advanced level of practice and/or developed an acknowledged advanced nursing practice role underpinned by graduate education [6]. In the absence of APN competencies specific to Kenya, therefore, the international competency framework developed by the International Council of Nurses [2] offered a useful reference point for the INEPEA initiative. In this framework, APN competencies are categorised within the three domains of (a) professional, ethical and legal practice; (b) care provision and management and (c) professional, personal and quality development. In the interest of public protection and workforce planning, the ICN offer member countries guidance on the scope of practice of an APN, as outlined in Table 1.

**Table 1:** **APN Scope of Practice** [2]**, p.13**

**APN practice requires:**	
•	Cognitive, integrative and technical abilities of the qualified nurse to put into practice ethical and culturally safe acts, procedures, protocols and practice guidelines;
•	The nurse to deliver evidence based care in primary, secondary and tertiary settings in urban and rural communities;
•	Practice of a high level of autonomy in direct patient care and management of health problems including case management competencies;
•	Accountability for providing health promotion, patient and peer education, mentorship, leadership and management of the practice environment;
•	Maintenance of currency and improving nursing practice achieved through the translation, utilisation and implementation of meaningful research;
•	The nurse to engage in partnerships with patients and health team members for determining resources needed for continuous care as well as partnering with stakeholders in influencing the policy that directs the health care environment.

The ICN’s account of advanced practice nursing shares much in common with other frameworks and models to be found in the international literature. For example, Mantzoukas and Watkinson’s review [11] identified seven generic features of advanced nursing practice: the use of knowledge in practice; critical thinking and analytical skills; clinical judgement and decision-making skills; professional leadership and clinical inquiry; coaching and mentoring skills; research skills and the skills for changing practice. This list does not tie advanced level practice to any particular role or job title, supporting the ICN’s contention that APN knowledge and competence is shaped by the needs of the particular context. However, it is notable that Mantzoukas and Watkinson’s review [11] concludes that the common goal of advanced nursing practice is ‘the attainment of practice autonomy and professional integrity within the nursing discipline so as to improve the provision of care’ (p.35). Their conclusion reflects the emphasis the ICN guidance places on a high level of autonomy in direct patient care, including the practical skills associated with the management of a clinical caseload.

While often treated uncritically, the significance of ‘autonomy’ in delineating the scope of APN practice can be questioned. For example, one reason for considering autonomy to be the hallmark of advanced nursing practice may be its origins in primary care. The primary care APN role, in particular, expands nursing into the traditional medical domains of examination, diagnosis and the management of discrete episodes of care, including prescribing rights. This is often considered to be a defining feature of the APN role within western countries, but may or may not be of significance in the African context. It is therefore important, as stated within the ICN definition, to consider the social and economic context for advanced nursing; it is to the Kenyan context that this paper now turns.

### The Kenyan context

Kenya is a low-income country situated on the east coast of Africa. Almost 80% of its 40 million people live in rural areas. With a Gross Domestic Product (GDP) per capita of $1800, Kenya ranks as the 198^th^ poorest of the 228 countries of the world according to 2012 estimates [12].

**Figure 1:**
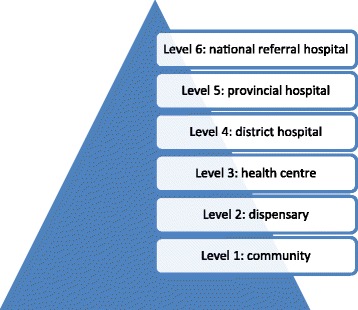
**The six levels of health care service delivery in Kenya.**

Given Kenya’s proportional spend of GDP on health care of 4.8% [12], service delivery represents a challenge, particularly in the arid northern areas. Kenya has a pluralistic health sector consisting of private and non-governmental providers operating alongside the public sector in which service delivery is organised according to 6 levels from primary to tertiary care (see Figure 1) [13].

Kenya has an acute shortage of doctors and nurses (0.14 doctors and 1.2 nursing/midwifery personnel per 1000 population [14]), as compared to a World Health Organisation (WHO) recommendation of a minimum of 2.5 nurses per 1000 population [15]. The shortage of health care personnel has been attributed to a number of factors, including a lack of workforce planning; high attrition of the health workforce; international and internal migration; the impact of HIV/AIDS and chronic under-investment in human resources for health [15]. As in many sub-Saharan African countries, Kenya has attempted to address the shortage of physicians by developing a mid-level cadre of non-physician clinicians known as ‘clinical officers’. They receive three years training and are able to diagnose, prescribe and generally manage the care of patients [16].

Nurses and midwives in Kenya can be found working in the enrolled or registered grades. Enrolled nurses work at the level of dispensaries and above, registered nurses in health centres and above. Registered nurses may hold either a diploma or a bachelor’s degree, with degree-qualified nurses generally found in the provincial and referral hospitals. A general registered nurse may train for a further year to become a registered midwife and community health nurse, or can undertake a further year of training in a range of clinical specialities. Enrolled nurses, who form 85% of the total nursing work force [17], can follow an education programme to convert to registered nurse status. There are also opportunities for diploma-qualified registered nurses to study for a post-registration degree. Enrolled and registered nurses with 5 or more years of experience can set up in private practice as independent health care providers [18]. Within the overall workforce, the distribution of nurses is skewed towards secondary care services: 71.6% work in hospitals, 13.2% in health centres and 15.2% in dispensaries [15]. Bearing in mind that the ICN consider a master’s degree the appropriate preparation for an APN, three Kenyan universities currently offer a masters degree in nursing [19].

At the time of the INEPEA project there was great enthusiasm expressed for advanced nursing practice by Kenyan nurse leaders and educators. However, within the context described above, it was unclear as to whether and how APN roles could be developed and implemented in the East African context, and the extent to which existing senior nursing roles could be considered ‘advanced’. The study reported here was therefore designed to explore the existing situation in Kenya, with reference to internationally recognised frameworks for advanced practice.

## Methods

This study adopted an exploratory qualitative design based on semi-structured interviews with ten nurses. The interviews began with an initial generative question - *Can you tell me about your first job as a nurse, and the subsequent development of your career?* This question was designed to elicit a wide-ranging narrative, followed by more focused questions related to advanced nursing [20]. Participants were asked to reflect upon (a) their professional and educational backgrounds and (b) the scope, responsibilities and challenges of their current role. A 12 item check-list of typical APN activities was developed based on the domains of practice described by the ICN [2] and other international literature (Table 2). The check-list was used as a prompt to encourage participants to reflect upon their day-to-day practice.

**Table 2:** **Check-list of typical APN activities** [2,11,24]

1.	Direct delivery of expert care to patients, families and/or communities as part of a health care team (assessing, planning implementing and evaluating nursing care; health promotion)
2.	Managing own, independent case load of patients: autonomous practice (assessing, diagnosing, planning and implementing treatment programmes, evaluating outcomes)
3.	Advising other health workers on direct care (acting as a consultant)
4.	Advocating for policy and practice improvement with managers and/or MoH
5.	Teaching colleagues and students in the practice area
6.	Formal teaching in the classroom setting
7.	Spending time in the library or online to identify evidence for practice
8.	Collecting data on service outcomes for audit and evaluation
9.	Formulating protocols and guidelines for the service
10.	Carrying out primary research as lead investigator
11.	Carrying out primary research as team member
12.	Administration (managing staff and resources, maintaining records)

Study participants were recruited by faculty of the Aga Khan University, Nairobi (the lead institution for the INEPEA project), and were purposively selected on the basis that they were working clinically at a level that might be considered advanced in the Kenyan context. They were selected to represent a range of clinical backgrounds, levels of service provision and rural and urban practice locations (Table 3). Government, private and not-for-profit (NFP) institutions were also represented. The majority of participants were graduates of the Aga Khan University’s post-registration Bachelor of Science in Nursing (BScN), the ‘Advanced Nursing Studies’ programme. In addition, and as further discussion about the nature of advanced practice took place between the UK and Kenyan faculty, it became clear that it was also important to speak with nurses practicing independently as private practice nurses (PPNs). Their scope of practice appeared to embrace many of the activities generally held to indicate advanced practice in the international context, although higher level nursing education for the PPN role is limited and their role would not generally be considered ‘senior’.

**Table 3:** **Characteristics of interview participants**

**Role & nursing qualification**	**Setting: community**	**Setting: hospital**	**Setting: urban**	**Setting: rural**
1. Nursing officer (BScN)		District hospital (Level 4)		x
2. Head nurse/ research nurse (BScN)	Research-funded primary care clinic (Level 2)		x	
3. Nursing officer (BScN)		National referral hospital (Level 6)	x	
4. Shift leader (BScN)		Private/NFP hospital (Level 6)	x	
5. District public health nurse (BScN)		District hospital (Level 4)		x
6. Shift leader (BScN)		Private/NFP hospital (Level 5)	x	
7. Sister-in-charge (BScN)		NFP/mission hospital (Level 4)		x
8. Head nurse (BScN)	Outreach clinic (Level 3)		x	
9. Private practice nurse (Dip Nursing)	Independent private practice (Level 3)		x	
10. Private practice nurse (Dip Nursing)	Independent private practice (Level 3)		x	

Ethical approval for the study was secured from the Aga Khan University as a follow-up to a situational analysis conducted as part of the INEPEA project [1]. All nurses were contacted in advance and invited to participate in the study by a member of the administrative staff of the Aga Khan University. Interviews were conducted in the participant’s own workplace, with the UK faculty as the main interviewer and the AKU faculty as liaison and support. Each participant was given an information sheet and asked to sign a consent form. With permission, interviews were recorded using a digital recorder and later transcribed in full.

### Data analysis

A thematic analysis of the interview transcripts was conducted using the steps advocated by Charmaz [21]:

1. initial coding, where the transcripts are read closely, line-by-line, and the researcher asks questions such as ‘what is happening here?’
2. selective (focused) coding, where the researcher uses frequently reappearing initial codes to sort and synthesise the data;
3. the development of categories (themes) and analytic frameworks from the focussed codes.

The initial coding stage was undertaken by the first author and produced 18 codes, generally reflecting the topics in the interview schedule but also revealing new, unanticipated topics of concern to the participants. Following selective coding, the number of codes was reduced to 12. In order to enhance rigour of the analysis and the credibility of the findings [22], the codes were reviewed and agreed by all four authors. The team collectively identified three key themes of significance in the debate concerning the context for advancing nursing practice in the Kenyan context:

- scope and activities of daily practice;
- contexts that constrain nurses from acting autonomously;
- contexts that facilitate autonomous practice.

## Results

All interview participants were female, senior, experienced nurses with an average of 20 years experience (ranging from 9 to 34 years). Five of the 10 participants were dual qualified as both nurse and midwife, and 8 were graduates of the Aga Khan Advanced Nursing Studies programme. Of the private practice nurses, one held a bachelor’s degree in community development and a master’s degree in public health (not mandatory for her practice); the other held a diploma-level qualification.

### Scope and activities of daily practice

**Figure 2:**
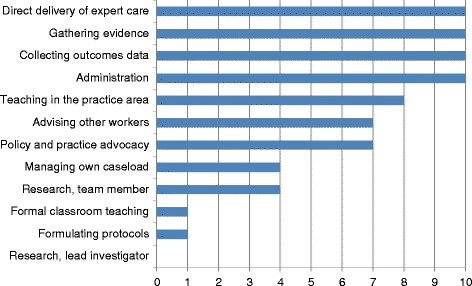
**Typical APN activities as demonstrated in nursing roles.**

As was a precondition for inclusion in this study, all interview participants were engaged in the direct delivery of expert care to patients, families and/or communities as part of a health care team. We also found that all 10 participants were engaged in collecting service data for audit and evaluation and a variety of other administrative duties including resource management, record keeping, rostering and bed allocation (Figure 2).

As might be expected of a group of degree-level senior nurses, the majority of BScN qualified participants were involved in teaching in the practice area, advising other health workers and advocating for policy and practice improvement. However, only one of the nurses interviewed contributed to formulating protocols and guidelines for their service area, or to classroom teaching in their specialist field.

All participants reported that they were able to access the internet and/or local libraries to obtain evidence for practice, thus utilising research findings in practice. However, none of those interviewed were leading research projects (including Participant 2, who was involved in the implementation rather than leadership of research).

During the interviews, we asked participants to describe their typical day. For the provincial and referral hospital nurses, a typical day was instantly recognisable to the UK researchers and is arguably typical for hospital nurses the world over. The day began with taking report from the night nurses, allocating staff to the appropriate areas, reviewing bed availability, overseeing admissions and discharges and accompanying doctors on their rounds. Depending on whether the participant was leading the shift, they would take on the clinical care of a group of patients and organise that care according to the principles of the nursing process. As senior nurses, they also reported having to attend many meetings.

Participants working in rural and primary care settings described a large public health element to their roles as a result of the poor living conditions of their populations:

*Just down here, this is a slum area, so the kind of clients we will have are those coming from low socio economic background, with conditions like TB. We have a lot of malaria. Recently we had a cholera outbreak, which is already under control. Children with respiratory tract infections, which is probably because of the dust and the kind of environment. There is no proper drainage or sewer.* (Participant 1)

In the rural areas, an APN activity such as advocating for policy and practice improvement could take on a different guise to that which might be expected in more affluent countries. Participant 5, for example, was a District Public Health Nurse working in a remote village about four hours from Nairobi. As a BScN Advanced Nursing Studies graduate, she was keen to keep her own knowledge and expertise up to date, and also to enable local people to connect to the wider world. She therefore lobbied one of the national mobile phone networks to erect a telecoms mast, thus enabling local people to make phone calls and equipping the rural district hospital with access to the internet: ‘*we really have to be proactive’*.

On the whole, and despite the challenging conditions, the BScN nurses we spoke to enjoyed their jobs. Despite their seniority, they drew the greatest satisfaction from hands-on care and making a difference in their patients’ lives:

*Personally, as an individual, I like being there. When a patient comes, particularly, a complicated case, or a very ill child, I want to work with that child throughout their stay here. The junior nurse may do the vital signs, the nurse may do this and that, but I’ll be there. So you find that at one point, I also do something. My role does not mandate that I do that, but there’s no way I can stay away from it.* (Participant 8)

To summarise, the participants in this study were undertaking many of the activities associated with the advanced practice role as outlined in Table 2. However, we found that none of the BScN nurses were acting with the degree of autonomy required to manage their own caseloads, often considered a hallmark of advanced nursing practice [11]. The factors constraining the degree-qualified nurses’ autonomy will be considered in the next section.

### Contexts that constrain nurses from acting autonomously

As noted above, the role of the senior nurses in hospital could be recognised as the role of the acute care nurse the world over. To some respondents, this role offered a considerable degree of autonomy in the management of their department:

*I have a lot of autonomy - with the patients who come here, I have a lot of say. In this trauma theatre, we decided we can only see trauma patients - gun shots, stab wounds, fractures, clean cases. Anything infectious, we don’t do.* (Participant 3)

However, in relation to clinical decision-making, the hospital nurses reported that their practice was limited by the traditional doctor-nurse role hierarchy. An interesting account was given by Participant 4, whose role was that of shift leader (senior staff nurse). She was highly experienced, having worked on the neonatal unit for 18 years and having completed a six week neonatal intensive care course overseas. However, in the absence of an advanced practitioner role, even experienced nurses had to defer to, or cover for, doctors. Participant 4 described her frustration thus:

*I cannot take a pen and prescribe a fluid, for example, but I know how to do it. So I can assist him to do it, but he’s the one to write it down.*

Participant 4 explained that the regulatory context in Kenya allows qualified nurses to prescribe from a limited range of medicines in particular circumstances, but this would not generally extend to an acute care hospital environment.

Other hospital nurses expressed their frustration at the slow pace of change and the power of the medical profession. For example, Participant 7 was a senior nurse working in the maternity unit of a mission hospital on the outskirts of Nairobi. She had worked in several other African countries, often with limited medical back up, and completed the BScN Advanced Nursing Studies in 2008. She was keen to base practice on evidence, for example, arguing against the need for pubic shaving when women are in labour. She described the process of trying to change practice as frustrating, with resistance from both medical and nursing colleagues:

*My biggest struggle, or experience of resistance, will be if you come up with an idea - dealing with the traditional way of doing things is always hard. You know the doctors, they have their own mindset and they have what they have learnt. OK, it’s also in us, it’s not only the doctors - we also prefer to do things the way we’ve always done them.*

Thus, the interviews suggested that there were some areas of conflict at the interface between nursing and the medical profession. Within a hospital context, it appeared that nurses were expected to defer to the expertise of doctors and had limited autonomy in clinical decision-making. Even in the two rural district hospitals, the BScN qualified nurses were not managing caseloads with the degree of autonomy that might be expected of an APN. In the cases of Participants 1 and 5, the clinical officers were making the initial contact with patients, examining, diagnosing and prescribing treatment. The dual qualified nurse/midwives, on the other hand, ran the maternity services. Participant 1 described the division of labour between nurses and clinical officers in her district hospital as follows:

*Mostly they* (clinical officers) *do the clerking of the patients. The nurses handle the midwifery, from the antenatal clinic all the way to the deliveries, the referrals and everything else. But the clinical officers handle the outpatients, clerking, seeing the sick adults and also seeing the sick under fives*. (Participant 1)

Participant 5 expressed a sense of frustration that nurses were not considered appropriate personnel to manage the rural hospitals – a role generally allocated to the clinical officer:

*It’s been a contentious issue, especially now that the nurses have got an opportunity to go and do a degree. But then again, there are policies. Maybe the policy will need to be changed to give an opportunity to nurses to become in-charges. Because in many cases the nurses do a marvellous job, they really run the facilities very well. You’d find that the clinical officer will come back to you again for consultation, ‘What do we do? What can we do here?*’ (Participant 5)

Given the hierarchical nature of health care professional roles in the Kenyan context, and the lack of a clinical career pathway, it is perhaps not surprising that few of the BScN nurses expressed a desire for further education in specialist or advanced nursing practice. Participant 4 was a notable exception, explaining that she would love to undertake further training in neonatal nursing. However, she noted the lack of opportunities for specialist nurses such as herself: *‘with nursing, I have come now to know that, when you are clinical, you might remain there for a long time, and your talents are not really noticed’.* Although the BScN nurses spoke very positively about the knowledge and confidence they had gained through studying for their Advanced Nursing Studies degree, only two wanted to undertake a master’s degree in nursing, as compared to four who aspired to a master’s in public health, one in research methods and one in international development.

### Contexts that facilitate autonomous practice

As noted earlier, with reference to Figure 1, degree-qualified nurses in Kenya are generally found in the provincial and referral hospitals (although our study included two BScN nurses working at Level 4 district hospitals). Given the emphasis of the International Council of Nurses’ and other international frameworks for APN on graduate education, the UK faculty expected to find the Advanced Nursing Studies BScN graduates to be working with higher levels of clinical autonomy. However, as data collection progressed and further discussion took place, it emerged that the cadre of nurses known as Private Practice Nurses (PPNs) in Kenya were practising with a degree of independence and autonomy that is often associated with APN in western countries.

Only two PPNs were interviewed during the course of this study due to its exploratory nature and time and resource constraints. However, the PPNs’ views and experiences are represented here as relevant to the consideration of contexts for advanced practice in Kenya. Both nurses maintained independent practices located in Nairobi. Participant 10 worked in an affluent suburb, serving mainly the employees of the richer households, and Participant 9 operated in a poorer area of the city. To gain a license to practice independently, nurses in Kenya must have at least five years’ experience and undergo regular inspection by the Ministry of Health. It is often enrolled nurses who set up in business; private practice is not considered appropriate for degree-educated nurses, who are expected to be in positions of leadership in secondary and tertiary care services.

However, the two PPNs we spoke to operated with a high level of independence and autonomy. In the case of the first clinic, the PPN (Participant 10) had a suite of rooms including a consulting room and a treatment room where minor surgical operations can be performed. She employed a technician and had a laboratory equipped to undertake simple tests, plus basic X Ray facilities. This PPN described a network of doctors she could call upon when a client needed a surgical or medical intervention, with gynaecology being the clinic’s specialism:

*In our minor theatre we do kind of a day surgery, where patients come for minor operations and are able to go home same day. We do dilatation and curettage, post abortion care for missed abortions or incomplete abortions. We do circumcisions for those who are to be circumcised, especially school/college children. We also do biopsies from the breast, from any other parts of the body, and we send the samples to Nairobi Hospital for histopathology, to detect whether it was cancer or just a benign growth. We also do minor skin operations, and stitching of wounds and cuts, and dressings, and burns management.*

It should be noted that the client’s initial appointment at the clinic was always with the PPN, who took a history and carried out a clinical examination, with the outcome being treatment in the clinic by the nurse or a physician/surgeon she employed, or referral on to the hospital if necessary. Participant 10 also ran a home care service, employing health care assistants to deliver care to clients in their home. A true entrepreneur, she was seeking the capital to establish a residential facility for older people, as Kenyan families are becoming increasingly fragmented, with adult children often working overseas. However, she was also committed to running a free clinic on an outreach basis in the local slum area and facilitating community development.

In contrast to the facilities of Participant 10’s clinic, Participant 9 had a very small consulting room in a much poorer area. Her practice was mainly based on the care of children, and she reported taking a holistic approach to disease management:

*So when the patient comes in, of course I’ll sit them down, usually we laugh and we have very good relationship, cordial relationships. I take the whole history about their sickness, whether there’s the same asthma in the family, and so on and so forth. I come up with a tentative diagnosis, then I take the observations, take their temperature, and check their blood, I check their chest, I auscultate the chest, and look at even general conditions of the child, and ask questions as I go along. And of lately I’ve had a lot of rickets, children have been having a lot of rickets. Malaria is rampant and it is so resistant.*

Private practice nurses are allowed to prescribe and dispense medicines, and Participant 9 kept a limited stock of common medicines, including antibiotics, which she sold on to her clients at a reasonable price. She did not make a great deal of money, as often clients could not afford to pay the full fee, but stated ‘*if I have medicine in my cupboard I will always give it*’. As well as a prescription, the client’s treatment programme may include a home visit for health promotion purposes, for example to advise a young mother on how to manage cooking with a kerosene stove so as not to further exacerbate her child’s respiratory illness.

Neither of the PPNs had a degree in nursing, although Participant 9 had a first degree in community development and a master’s degree in public health. In addition to running her practice, she lectured on community nursing at a local university. Participant 10, on the other hand, felt well prepared for private practice through nearly 20 years of experience with the flying doctor service (AMREF), working in remote areas and establishing clinics where there was no other health care provision. Both of the PPNs expressed great satisfaction with their work, which required them to keep up to date with regular continuing professional education and undergo biannual re-registration. Although both had good relationships with medical colleagues to whom they referred patients or whose services they engaged, both felt the overall relationship between doctors and nurses in Kenya could be improved, and that nurses need to gain confidence:

*Nurses have been so lax. They’ve just been waiting to be given instructions by doctors. And doctors have not been very kindly to nurses. They have made the nurses work even until they feel like they… they are second class.* (Participant 9)

*My colleagues who are just working in hospitals, they say, “You’re so bold that you can work alone.” But given my background at AMREF, I’ve never relied on somebody to give me direction.* (Participant 10)

It seems poignant that the two interview participants whose daily activities came closest to the ICN’s characterisation of the scope of practice for APNs in terms of a ‘high level of autonomy’ (Table 1) had the least requirement in terms of education and training in the Kenyan context. This is, perhaps, because PPNs are considered to operate at Level 1 or 2 of the Kenyan hierarchy of health care service delivery (Figure 1). However, we accept that our sample of only two PPNs may be atypical in terms of experience, expertise and (in the case of Participant 9), educational preparation. While PPN practice is closely regulated by the Ministry of Health, both PPNs interviewed for this study considered themselves unusually well qualified, with some other PPNs reportedly being of ‘*very low level’* (Participant 9).

## Discussion

As noted earlier in relation to conceptual frameworks for advanced nursing practice, the ICN definition emphasises the importance of APNs practising with autonomy in clinical decision-making and patient management. In the list of activities explored during the interviews, this area of APN practice was described as *‘Managing own, independent case load of patients: autonomous practice (assessing, diagnosing, planning and implementing treatment programmes, evaluating outcomes)’* (see Table 2). We found that only four of our respondents undertook this kind of autonomous practice. Two of the four were referring to their practice within maternity services as dual-qualified nurse/midwives, roles somewhat outside the scope of this discussion (Participants 1 and 7).

The two nurses who came closest to the international understanding of autonomous advanced practice nursing were the two private practice nurses (Participants 9 and 10). Ironically, therefore, we observed what appeared to be an inverse relationship between higher levels of nursing education and the degree of autonomy that characterises nursing practice in the Kenyan health care system. In this study, the nurses practising with the most autonomy were those working in the lower tiers of the primary care services. As noted earlier, nearly 72% of nurses in Kenya work in hospitals [15], with the better qualified graduate nurses to be found in the teaching and referral hospitals. Indeed, the majority of nurses are given their posts by the Ministry of Health: primary care and independent private practice is generally considered the province of enrolled and diploma-level registered nurses.

The shortfall of doctors in Kenya in the rural areas and district hospitals has been addressed (as in other African countries) by the development of the ‘clinical officer’ cadre. Although a shortage of doctors has been a driver for the emergence of the advanced nurse practitioner role in western countries, it seems this driver has not produced a similar outcome in Kenya. In part, this is because the potential for nurse-doctor substitution is undermined by an extreme shortage of qualified nurses as well as qualified doctors [14]. In addition, and unlike qualified doctors and nurses, clinical officers are relatively cheap to train and are not able to emigrate in search of better pay and working conditions as their qualification is not internationally recognised [23]. However, there has been a curious silence in the international literature on advanced practice about how this mid-level role potentially interfaces with the nursing profession. In Kenya, our data suggests that role tension can exist between experienced nurses and clinical officers within the district hospitals. Indeed, one might conclude that the existence of this cadre of health care workers renders the development of the APN role in primary care in Kenya unlikely. In Kenya, clinical officers undergo independent training and their role is not seen as aligned with, or developed from, the nursing profession. This is a professional boundary that requires further investigation if the development of APN roles is to proceed.

The private practice nurses, on the other hand, do seem to have been granted a license to address the shortfall of doctors in primary care within the Kenyan context. Their practice is regulated by the Ministry of Health, and a basic level of in-service training is provided. However, the PPNs do not fulfil the criteria set out by the ICN [2] in terms of the educational preparation for their role, and do not appear to exercise the APN sub-roles of educator, researcher and consultant and expert in practice development [24]. Indeed, autonomy in clinical practice is only one dimension of the APN role as described by the ICN [2], albeit a fundamental element of the nurse practitioner role [11]. Further research is needed into how private practice nurses are working in Kenya and other African countries and their levels of expertise, not least to attempt to quantify their impact on health outcomes.

It was encouraging to see the importance all nurses interviewed during the course of this study placed on evidence-based practice, and to find that the majority had internet and library access that allowed them to keep up to date. The nurses in the rural hospitals were able to exercise some control over their practice, particularly when working as midwives or delivering public health programmes. However, the senior nurses and midwives working in hospitals were to a large extent dependent on the support of medical colleagues and hospital managers to advance their practice. This reflects Manley’s observation [25] that advancing nursing practice depends not only on the individual nurse but also on the organisational context in which he or she practises.

According to Manley [25], the contextual pre-requisites for advancing practice include shared beliefs and values; open non-hierarchical management and sufficient organisational authority attributed to post. Hospital nurses in this study seemed to suffer from a hierarchical management structure and a lack of organisational authority, where deference to the doctor was expected. In addition, there was little opportunity to progress in a clinical or clinical-academic career as it seemed that specialist nursing expertise was not generally recognised and valued, and opportunities for nurses to engage in research were few. This may explain why few of our respondents were looking to undertake a nursing master’s degree as there was no obvious career pathway as a result other than becoming a nurse teacher. Undertaking a master’s degree in public health appeared a more attractive option, not least because it could lead to work with a higher paying and more prestigious non-governmental organisation. Alternatively, out-migration to countries with greater opportunities for professional development is an attractive option for Kenya’s younger, and most highly-educated, nurses [26].

## Conclusions

Analysis of the interviews suggests that, although purposefully selected because they were considered to be working at an advanced level, the BScN Advanced Nursing Studies graduates were not working as advanced practice nurses as defined by the ICN [2]. In Kenya, it seems that highly qualified nurses tend to move into management and education, and see little opportunity for advancement while remaining in clinical practice. We conclude, therefore, that the potential for the development of advanced nursing practice is constrained in part by structural health system factors congruent with Kenya’s low-income status, i.e. the shortage of well-qualified nurses and doctors, and the presence of an established clinical officer cadre. In addition, bachelors or masters level education seems to offer a route to ‘advanced’ expertise in management, leadership, education or advocacy roles but has limited potential to develop autonomous clinical practice in a context characterised by traditional, hierarchical doctor-nurse relationships.

However, in a region where the overall number of registered nurses is so low, it may be that the management, leadership, advocacy and educational functions of the advanced practice role are of greater importance, not least because senior nurses supervise others. There is a pressing need for advanced nurses who can implement evidence-based practice and exercise clinical leadership in the drive to attain improvements in quality of care and better patient outcomes [15]. Indeed, as part of an extensive consultation with regulatory bodies, ministries of health and other stakeholders, the INEPEA project concluded that competencies for APNs in the East Africa region should include domains such as ‘being knowledgeable and competent in gender mainstreaming and cultural awareness’ and ‘ethical decision-making and anti corruption’ [1], amongst other competencies more familiar to a western audience.

The potential contribution of advanced practice nurses to Kenyan health care goes beyond policies of task-shifting as advocated by the WHO [7], in this case where nurses substitute for doctors. Increased recognition, and support for, the practice development and leadership functions of advanced practice nurses, in addition to a strengthening of expertise in clinical practice, could reap dividends at all levels of the health care system described in Figure 1. In addition, a clinical career pathway leading to recognised APN roles could enhance job-satisfaction and retention among qualified nurses. As noted by McCarthy et al. [10], such a clinical career pathway would need to be supported by updated regulatory arrangements, requiring capacity building among nursing and midwifery regulators.

In their recent systematic review of advanced nursing roles, Jokiniemi et al. [24] conclude that a more balanced international description and framework for advanced nursing practice will be achieved when more countries take part in the discussion. While this study has limitations in terms of sample selection and size, it is hoped that it will contribute a much-needed African perspective to the international literature on advanced nursing.

## Abbreviations

APN: Advanced practice nurse
AKU: Aga Khan University
AMREF: African Medical and Research Foundation
BScN: Bachelor of Science in Nursing
CIA: Central Intelligence Agency
Dip: Diploma
GDP: Gross Domestic Product
INEPEA: Improving Nursing Education and Practice in East Africa
ICN: International Council of Nurses
MOH: Ministry of Health
MPH: Master of Public Health
NCAPD: National Coordinating Agency for Population and Development
NFP: Not for profit
PPN: Private practice nurse
UK: United Kingdom
WHO: World Health Organisation
